# Dopamine-Induced Apoptosis of Lactotropes Is Mediated by the Short Isoform of D2 Receptor

**DOI:** 10.1371/journal.pone.0018097

**Published:** 2011-03-25

**Authors:** Daniela Betiana Radl, Jimena Ferraris, Valeria Boti, Adriana Seilicovich, Dipak Kumar Sarkar, Daniel Pisera

**Affiliations:** 1 Facultad de Medicina, Instituto de Investigaciones en Reproducción, Universidad de Buenos Aires, Buenos Aires, Argentina; 2 Endocrine Program, Department of Animal Sciences, Rutgers, The State University of New Jersey, New Brunswick, New Jersey, United States of America; University of Windsor, Canada

## Abstract

Dopamine, through D2 receptor (D2R), is the major regulator of lactotrope function in the anterior pituitary gland. Both D2R isoforms, long (D2L) and short (D2S), are expressed in lactotropes. Although both isoforms can transduce dopamine signal, they differ in the mechanism that leads to cell response. The administration of D2R agonists, such as cabergoline, is the main pharmacological treatment for prolactinomas, but resistance to these drugs exists, which has been associated with alterations in D2R expression. We previously reported that dopamine and cabergoline induce apoptosis of lactotropes in primary culture in an estrogen-dependent manner. In this study we used an in vivo model to confirm the permissive action of estradiol in the apoptosis of anterior pituitary cells induced by D2R agonists. Administration of cabergoline to female rats induced apoptosis, measured by Annexin-V staining, in anterior pituitary gland from estradiol-treated rats but not from ovariectomized rats. To evaluate the participation of D2R isoforms in the apoptosis induced by dopamine we used lactotrope-derived PR1 cells stably transfected with expression vectors encoding D2L or D2S receptors. In the presence of estradiol, dopamine induced apoptosis, determined by ELISA and TUNEL assay, only in PR1-D2S cells. To study the role of p38 MAPK in apoptosis induced by D2R activation, anterior pituitary cells from primary culture or PR1-D2S were incubated with an inhibitor of the p38 MAPK pathway (SB203850). SB203580 blocked the apoptotic effect of D2R activation in lactotropes from primary cultures and PR1-D2S cells. Dopamine also induced p38 MAPK phosphorylation, determined by western blot, in PR1-D2S cells and estradiol enhanced this effect. These data suggest that, in the presence of estradiol, D2R agonists induce apoptosis of lactotropes by their interaction with D2S receptors and that p38 MAPK is involved in this process.

## Introduction

Dopamine (DA) is the predominant catecholaminergic neurotransmitter in the mammalian brain and is involved in a variety of functions such as locomotion, reinforcement, food intake, emotion and neuroendocrine secretion. In the anterior pituitary gland, DA inhibits prolactin (PRL) synthesis and release, as well as lactotrope proliferation [1]. In addition to these well established actions, we previously reported that DA induces apoptosis of lactotropes from female rats in an estrogen-dependent manner [2]. These pituitary actions are exerted through the D2 receptor (D2R), a member of the G protein-coupled receptor superfamily [3].

D2R exists as two alternatively spliced isoforms, long (D2L) and short (D2S). D2L differs from D2S by the presence of additional 29 amino acid residues within the third intracellular loop. D2L and D2S can couple to different G inhibitory proteins [4], [5] and, although both isoforms can transduce the intracellular signal correctly [4], it has been reported that D2S is more efficient for inhibiting adenylyl cyclase than D2L [6]. Both isoforms can be expressed in the same cell, but D2L is the main isoform present in the anterior pituitary, and estradiol (E2) was shown to increase the D2L/D2S ratio [7]–[9].

Cabergoline (CAB), a D2R agonist, is the most effective compound for pharmacological treatment of prolactinomas [10], strongly reducing PRL secretion and lactotrope proliferation [11], [12]. Although DA agonists have been proven to be successful in normalizing serum PRL levels, a subset of patients with prolactinomas does not respond to CAB, suggesting that D2R expression is altered. In fact, prolactinomas resistant to D2R agonist treatment have been shown to express less D2R mRNA than responsive tumors [13]. Moreover, some studies suggest that alterations in the proportion of D2L and D2S isoform expression could be involved in D2R agonist resistance [10], [14], [15]. In addition, estrogens sensitize anterior pituitary cells to different proapoptotic stimuli [16], [17], and we have observed that CAB induces apoptosis of lactotropes only when cells are cultured in the presence of E2 [2], making it plausible that the hormonal milieu could affect the action of D2R agonists in patients with prolactinomas.

D2R is coupled to distinct intracellular pathways including different MAPKs [18]. DA-induced apoptosis of neuroblastoma cells [19] and pituitary-derived GH3 cell line [20] involves p38 MAPK activation. The abnormal transduction of D2R signaling could also explain the failure of D2R agonist treatment in resistant prolactinomas [10].

In the present work, we studied the role of D2R isoforms, D2L and D2S in the apoptosis of lactotropes induced by DA. We also investigated the participation of p38 MAPK in this action. We confirm the influence of E2 in the proapoptotic action of CAB on anterior pituitary cells in an *in vivo* model.

Also, we show that DA induces apoptosis of lactotropes through D2S receptor activation in an E2-dependent manner and that p38 MAPK is involved in this action.

## Methods

### Ethics Statement

All procedures complied with the Ethical Committee of the School of Medicine, University of Buenos Aires and the NIH Guide for the Care and Use of Laboratory Animals.

### Drugs

All drugs, media and supplements were obtained from Invitrogen (Carlsbad, CA, USA), except Dulbecco's modified Eagle's medium (DMEM), bovine serum albumin (BSA), 17β-estradiol (E2), DA, normal horse serum and protease inhibitor cocktail (Sigma, St. Louis, MO, USA), fetal bovine serum (GBO, Buenos Aires, Argentina), SB203580 (Stressgen, PA, EEUU), gentamicin (Promega, Madison,WI), Vectashield (Vector Laboratories, Inc., Burlingame, CA, USA), all terminal deoxynucleotidyl transferase-mediated deoxyuridine triphosphate nick end-labeling (TUNEL) reagents (Roche Molecular Biochemicals, Mannheim, Germany), Cell Death Detection (ELISA^Plus^) (Roche Molecular Biochemicals, Mannheim, Germany), guinea pig rat prolactin antiserum (Dr. A. Parlow, National Hormone and Pituitary Program, Torrance, CA, USA), anti-guinea pig rhodamine-conjugated secondary antibody (Chemicon International, Temecula, CA, USA), fluorescein isothiocyanate (FITC) Annexin-V (BD Pharmingen, San Jose, CA, USA), anti-p38 MAPK and anti-phospho-p38 MAPK antibodies (Cell Signaling Technology, CA, USA) and cabergoline (kindly donated by Holliday-Scott, Buenos Aires, Argentina).

### Animals

Adult female Wistar rats were kept in controlled conditions of light (12 hour light-dark cycles) and temperature (20–25°C). Animals were fed standard lab chow and water *ad libitum*. The rats were ovariectomized (OVX) under ketamine (75 mg/kg, i.p.) and xylazine (10 mg/kg, i.p.) anesthesia. For primary cultures, the rats were euthanized by decapitation 15 days after ovariectomy and anterior pituitary glands were removed. For *in vivo* experiments, the rats were s.c. implanted in the skin of the back with silicone capsules containing 1 mg of 17β-estradiol (E2) or empty capsules (OVX) at the time of ovariectomy. The capsules (length 20 mm, outer diameter 2 mm) were prepared in the laboratory and were filled with 50 µl of E2 solution (20 mg/ml) diluted in ethanol. When ethanol was evaporated the capsules were sealed with a silicone adhesive. These capsules induce plasma levels of E2 similar to those of rats at proestrus [21]. After 15 days of ovariectomy, the rats were injected with cabergoline (CAB, 1 mg/kg, i.p.) or 0.9% NaCl (CONTROL). After 16 h, the rats were euthanized, anterior pituitary glands obtained and cells dispersed to analyze apoptosis by Annexin-V staining.

### Primary culture of anterior pituitary cells

A pool of anterior pituitary cells from 5–8 OVX rats was used for each primary culture. Anterior pituitary glands were washed several times with DMEM supplemented with 10 µl/ml MEM amino acids, 2 mM glutamine, 5.6 µg/ml amphotericin B, 25 µg/ml gentamicin (DMEM-S) and 3 mg/ml BSA. Then the glands were cut into small fragments and dispersed enzymatically by successive incubations in DMEM-S with BSA, containing 0.75% trypsin, deoxyribonuclease type I (DNase; 45 U/ml) and 10% fetal calf serum previously treated with 0.025% dextran-0.25% charcoal (FCS) to remove steroids. The cells were then washed with Krebs-Ringer bicarbonate buffer free of Ca^2+^ and Mg^2+^ (KRBCMF), pH 7.4. Finally, the cells were dispersed in KRBCMF by extrusion through a Pasteur pipette. Dispersed cells were washed twice and resuspended in DMEM-S with 10% FCS. Cell dispersion yielded about 3–3.5×10^6^ cells/gland. Cell viability as assessed by trypan blue exclusion was over 90%. The cells were seeded onto coverslides in 24-well tissue culture plates (1×10^5^ cells/ml/well) and cultured in DMEM-S with 10% FCS for 24 h. Then the cells were cultured in fresh medium containing 17β-estradiol (E2, 1 nM) for 24 h and in the same media without serum for a further 24 h. After this period, cells were incubated in the same media containing different drugs according to each experiment.

### Cell lines

The PR1 cell line was derived from a pituitary tumor of a Fischer-344 ovariectomized rat treated with E2 for 3 months [22]. These cells do not express D2Rs [23] and secrete PRL [22]. Stably transfected PR1 cells with an expression vector containing cDNA encoding D2L (PR1-D2L), D2S (PR1-D2S) receptors or an empty vector (PR1-V) [23] were maintained in a 1∶1 mixture of Dulbecco's modified Eagle's medium and Ham's F-12 medium (DMEM/F-12; Sigma) containing 10% FBS previously treated with 0.025% dextran-0.25% charcoal and 800 µg/ml gentamicin. The cells were seeded onto coverslides in 24-well culture plates (1×10^5^ cells/ml/well) for the TUNEL method, in 24-well culture plates (5×10^5^ cell/ml/well) for ELISA or in 6-well plates (1×10^6^ cells/ml/well) for protein extraction. Then, the cells were maintained for 24 h in DMEM/F-12 supplemented with human transferrine (100 µM), insulin (5 µM), putrescine (1 µM) and sodium selenite (30 nM) without serum (DMEM/F-12-SS). Finally, the cells were cultured with vehicle (VEH, ethanol, 1 µl/l) or E2 (1 nM) in DMEM/F-12-SS for 48 h and incubated in the same media containing different drugs according to each experiment.

### Detection of Apoptosis by FITC Annexin-V Staining

Anterior pituitary glands from *in vivo* experiments were dispersed with trypsin/DNase as previously described [2]. Then the cells were washed in cold PBS and resuspended in binding buffer (10 mM HEPES/NaOH, pH 7.4, 140 mM NaCl, 2.5 mM CaCl_2_). The cells were incubated with 5 µl FITC Annexin-V and 10 µl propidium iodide (PI, 50 µg/ml) for 15 min in darkness. Cells were immediately analyzed by flow cytometry (Becton-Dickinson FACScalibur) and data analyzed by WinMDI98 software. Annexin-V-positive/PI-negative cells were considered early apoptotic cells, whereas Annexin-V-negative/PI-positive cells were considered necrotic cells. Double-positive (Annexin-V-positive/PI-positive) cells were considered to be in a late stage of apoptosis.

### Detection of Apoptosis by Cell Death Detection (ELISA^PLUS^)

The apoptotic response of PR1 cells was measured by Cell Death Detection (ELISA^Plus^). This kit is used for relative quantification of histone-complexed DNA fragments (mono- and oligonucleosomes) induced by apoptosis. After treatment, cells were lysed to obtain the nucleosomes and processed according to the manufacturer's protocol. Optic density (OD) was measured in a spectrophotometer at 405 nm.

### Detection of Apoptosis by TUNEL assay

After the culture period, the cells were fixed with 4% paraformaldehyde for 10 min and permeabilized by microwave irradiation. DNA strand breaks were labeled with digoxigenin-deoxyuridine triphosphate using terminal deoxynucleotidyl transferase (0.18 U/µl) according to the manufacturer's protocol. After incubation with 10% horse serum, slides were incubated for 1 h with antidigoxigenin-fluorescein antibody (aDIG, 1∶10) to detect incorporation of nucleotides into the 3-OH end of damaged DNA. Slides were mounted with Vectashield (Vector Laboratories, Inc., Burlingame, CA, USA) containing 4,6 diamidino-2-phenylindoledihydrocloride (DAPI) for DNA staining and visualized in a fluorescence light microscope (Axiophot; Carl Zeiss Jena, Germany). In primary culture of anterior pituitary cells, lactotropes were identified by immunofluorescence. After incubation with 10% serum in PBS, the cells were incubated for 1 h with guinea pig rat prolactin antiserum (1∶1.500). Then, the slides were incubated for 1 h with aDIG (1∶10) and a rhodamine-conjugated anti-guinea pig secondary antibody (1∶200). The percentage of total apoptotic cells was calculated as (TUNEL-positive cells/total anterior pituitary cells)×100. The percentage of apoptotic lactotropes was calculated as (TUNEL-positive prolactin-immunoreactive cells/prolactin-immunoreactive cells)×100. The percentage of lactotropes determined by immunofluorescence was between 25–40% of total anterior pituitary cells.

### Western Blot

Total proteins were extracted from cultured cells in lysis buffer containing protease inhibitor cocktail and phosphatase inhibitors (10 mM NaF, 2 mM Na-orthovanadate, 80 mM β-glycerol phosphate). The protein concentration of each sample was determined by Bradford protein assay (BioRad Laboratories, CA, USA). Twenty µg of total proteins were size-fractionated in 12% SDS-polyacrylamide gel and then electrotransferred to polyvinyl difluoride (PVDF) membranes. Blots were blocked for 2 h in 5% nonfat dry milk-TBS-0.1% Tween 20 at room temperature and incubated overnight with the appropriate primary antibody in the same buffer at 4°C. The primary antibodies used were: anti-p38 MAPK (1∶1000) and anti-phospho-p38 MAPK (1∶2000). Then, membranes were incubated for 1 h at room temperature with the corresponding peroxidase conjugated secondary antibody. Immunoreactivity was detected by enhanced chemiluminescence. Membranes were exposed to X-ray films and developed using an X-Ray developer.

### Statistical Analysis

Each experiment was performed at least twice. The percentage of apoptotic cells determined by flow cytometry was expressed as mean ± SE and evaluated by two-way ANOVA followed by Tuckey's test. The optic density measured by ELISA was expressed as mean ± SE and analyzed by one-way ANOVA followed by Dunnett's test. The number of apoptotic cells identified by TUNEL was determined in duplicate slides from independent experiments. Results were expressed as the percentage ±95% confidence interval (CI) of apoptotic cells of the total number of cells counted in each specific condition. Differences between proportions were analyzed by χ^2^ test [2]. Differences were considered significant if p<0.05.

## Results

### Effect of CAB on anterior pituitary cell apoptosis

**Figure 1:**
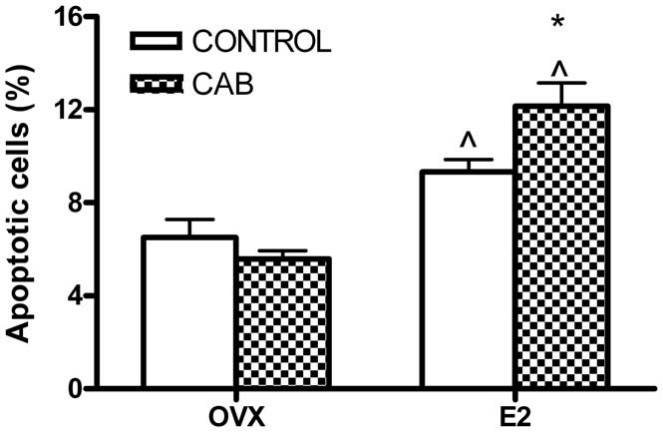
CAB induces apoptosis of anterior pituitary cells in an E2-dependent manner. OVX and E2 treated rats were injected with CAB (1 mg/kg, ip) or vehicle (CONTROL) and euthanized 16 h later. Anterior pituitary cells were dispersed and apoptosis was detected by Annexin-V and flow cytometry. Each column represents the mean ± SE of the percentage of apoptotic cells (n = 8 rats/group). Data were analyzed by two-way ANOVA, followed by Tuckey's test. *p<0.05 vs. respective control without CAB, ∧p<0.05 vs respective control without E2.

To confirm the permissive action of E2 on the apoptotic action of D2R activation in an *in vivo* model, apoptosis in the anterior pituitary gland was evaluated in OVX and E2-treated rats injected with CAB (determined by FICT Annexin-V staining). As previously described [24], E2 treatment per se increased apoptosis of anterior pituitary cells. Whereas CAB did not modify the percentage of anterior pituitary apoptotic cells from OVX rats, this D2R agonist induced apoptosis in the pituitary from E2-treated rats (Fig. 1).

### Effect of DA on the apoptosis of lactotropes expressing D2L or D2S receptor isoforms

**Figure 2:**
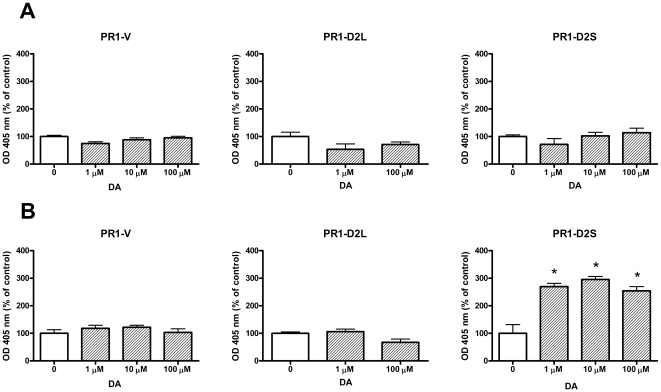
DA increases apoptosis in PR1-D2S cells incubated in the presence of E2. PR1-V, PR1-D2L or PR1-D2S cells were cultured with DMEM-F12-SS in the absence (A) or presence of E2 (1 nM, B) for 48 h. Then the cells were incubated with DA (1 µM-100 µM) in the presence or absence of E2 for 4 h and apoptosis was detected by ELISA. Each column represents the mean ± SE expressed of OD as the percentage of control without DA (n = 5 wells/group). Data were analyzed by ANOVA followed by Dunnett's test. * p<0.05 vs control without DA.

**Figure 3:**
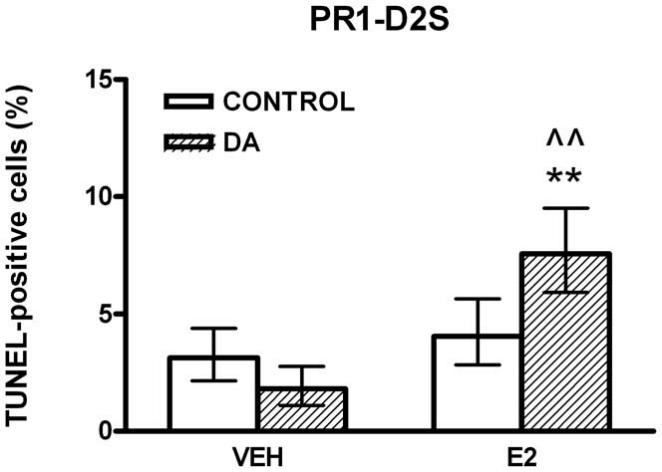
DA increases the percentage of apoptotic PR1-D2S cells incubated in the presence of E2. PR1-D2S cells were cultured with DMEM-F12-SS in the absence (VEH) or presence of E2 (1 nM) for 48 h. Then the cells were incubated with DA (1 µM) with or without E2 for 4 h. Each column represents the percentage of TUNEL-positive cells ± CI (>2,000 cells/group). Data from at least two separate experiments were analyzed by χ^2^. ** p<0.01 vs respective control without DA. ∧∧ p<0.01 vs respective control without E2.

To study the role of D2R isoforms in the apoptosis of lactotropes induced by DA, we evaluated the proapoptotic action of this catecholamine on PR1 cells expressing either D2L or D2S. Considering that DA-induced apoptosis of lactotropes is E2-dependent [2], PR1 cells were incubated either in the presence or absence of E2 (1 nM). DA did not modify the apoptosis (determined by an ELISA assay) in PR1-V and PR1-D2L cells incubated either in the absence or presence of E2 (Fig. 2A and B). Whereas this catecholamine did not modify the apoptosis of PR1-D2S cells incubated in the absence of E2 (Fig. 2A), DA induced apoptosis of these cells when they were cultured with E2 (Fig. 2B). We also analyzed the apoptotic action of DA in PR1 cells by TUNEL assay. DA did not modify the percentage of TUNEL-positive PR1-V and PR1-D2L cells incubated in the absence or presence of E2 (data not shown). However, DA increased the percentage of TUNEL-positive PR1-D2S cells only when these cells were incubated with E2 (Fig. 3).

### Involvement of p38 MAPK on DA-induced apoptosis

**Figure 4:**
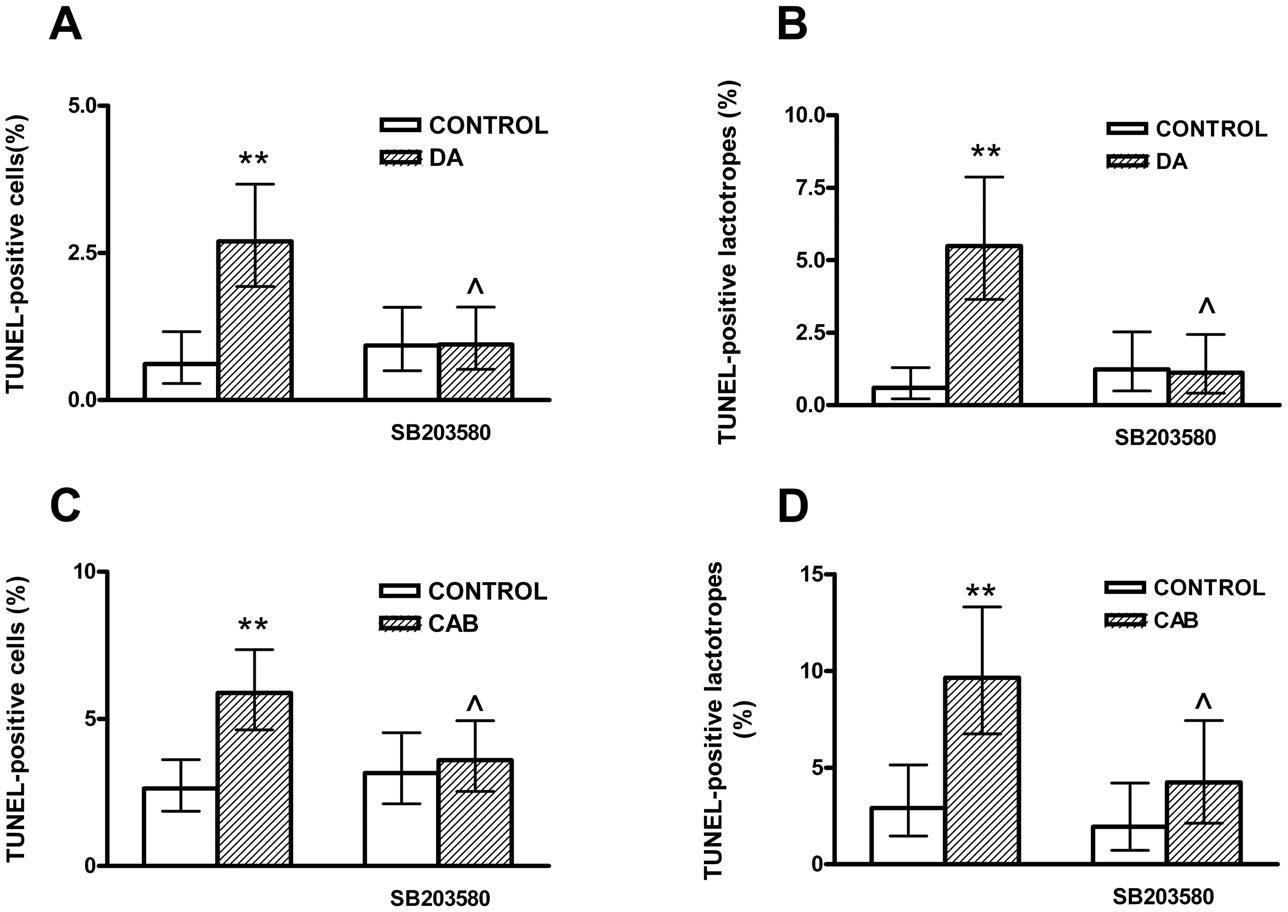
A p38 MAPK inhibitor blocks the apoptosis of anterior pituitary cells induced by D2R activation. Anterior pituitary cells were cultured with E2 (1 nM) for 48 h. Then the cells were preincubated with SB203580 (1 µM), a p38 MAPK inhibitor, for 30 min and then incubated with DA (1 µM, A and B) or CAB (1 µM, C and D) in the presence or absence of SB203580 for 4 h. Each column represents the percentage of TUNEL-positive cells ± CI (>2,000 cells/group, A, C) or the percentage of TUNEL positive lactotropes ± CI (>500 cells/group B, D). Data from at least two separate experiments were analyzed by χ^2^. ** p<0.01 vs respective control without DA or CAB. ∧ p<0.05 vs respective control without SB203589.

**Figure 5:**
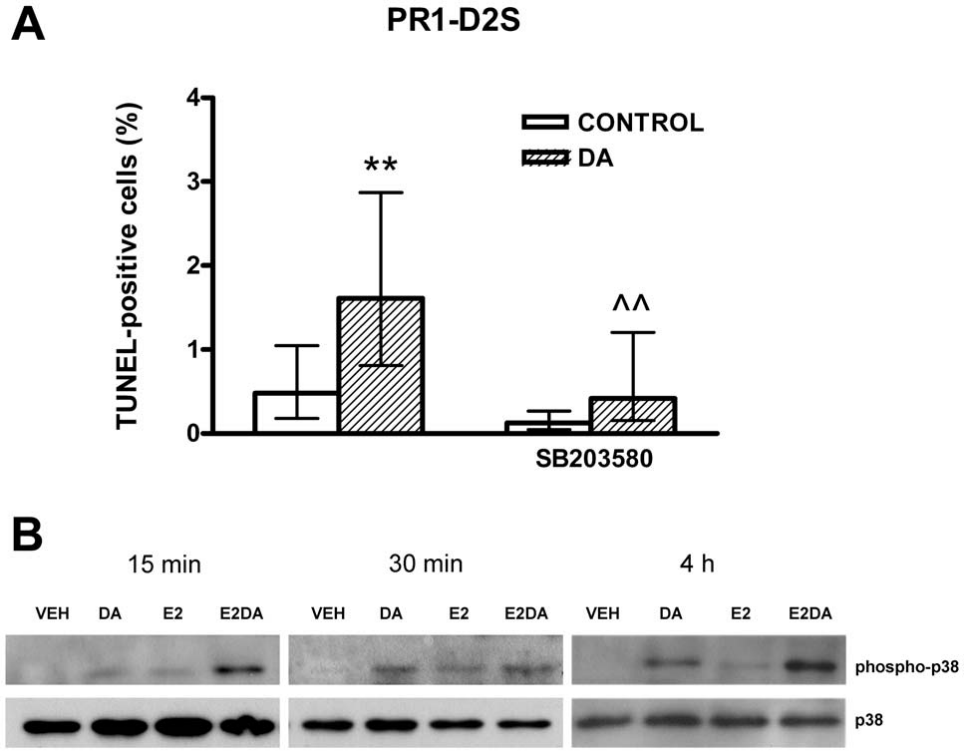
p38 MAPK is involved in DA-induced apoptosis of PR1-D2S cells. A: PR1-D2S cells were cultured with E2 (1 nM) for 48 h. Then the cells were preincubated with SB203580 (1 µM), a p38 MAPK inhibitor, for 30 min and then incubated with DA (1 µM) in the presence or absence of SB203580 for 4 h. Each column represents the percentage of TUNEL-positive cells ± CI (>2,000 cells/group). Data from at least two separate experiments were analyzed by χ^2^. ** p<0.01 vs respective control without DA. ∧∧ p<0.01 vs respective control without SB203580. B: PR1-D2S cells were cultured with or without E2 (1 nM) for 48 h. Then the cells were incubated with DA (1 µM) in the presence of E2 or VEH for 15 min, 30 min or 4 h. Proteins were extracted and p38 MAPK and phospho-p38 MAPK were detected by western blot.

To investigate whether p38 MAPK is involved in the apoptosis of anterior pituitary cells induced by D2R activation, anterior pituitary cells from OVX rats cultured with E2, were incubated with DA or CAB in the presence of SB203580, a p38 MAPK inhibitor. SB203580 blocked the apoptotic effect of either DA or CAB in anterior pituitary cells and lactotropes from primary cultures (Fig. 4). Also, in PR1-D2S cells cultured with E2, the apoptotic effect of DA was reverted by SB203580 (Fig. 5A). In addition, we determined the effect of DA on p38 phosphorylation in PR1 cells by western blot. DA did not modify p38 phosphorylation in PR1-V and PR1-D2L cells incubated either in the absence or presence of E2 (data not shown). However, DA induced p38 MAPK phosphorylation in PR1-D2S cells, an effect that E2 seems to enhance (Fig. 5B).

## Discussion

DA is the main inhibitor of lactotrope function and its interaction with D2R not only inhibits PRL secretion, PRL gene expression and lactotrope proliferation [1], but also induces apoptosis of these cells [2]. D2R knock-out (KO) mice and mice lacking the ability to synthesize DA show pituitary hyperplasia and hyperprolactinemia [25]–[27]. Two isoforms of D2R are synthesized by alternative splicing, D2S and D2L [1]. However, no specific ligands exist allowing to discriminate D2S from D2L actions. Therefore, KO animal models for D2L (D2L−/−) [28], [29] or animals with D2R isoforms overexpression [30] have been developed. Also, cell lines expressing D2L or D2S isoforms have been generated [20], [23], [31]. In GH3, a somatolactotrope cell line lacking D2R, D2L and D2S were described to mediate dopaminergic repression of PRL gen promoter when cells are transfected with these isoforms [32]. However, whereas D2R−/− female mice are hyperprolactinemic [26], [27], D2L−/− female mice exhibit normal serum PRL levels [29]. Since D2L−/− mice express D2S, it has been suggested that D2S can substitute the function of D2L in the control of PRL secretion [29]. However, it is possible that only the D2S isoform is involved in the inhibition of PRL release induced by DA. In fact, Iaccarino et al. demonstrated that mice overexpressing D2S receptor show a reduction in pituitary size, lactotrope number, PRL mRNA expression and serum PRL levels compared to WT mice and D2L overexpressing mice [30]. Also, DA was reported to exert an antiproliferative action in PR1 cells expressing D2S receptor but not in PR1 cells expressing the D2L isoform [23]. Both isoforms are expressed in the anterior pituitary gland [1]. Even though the predominant one is D2L [7]–[9], we show that DA induces apoptosis of PR1-D2S cells, whereas D2L activation does not seem to be implicated in DA proapoptotic action. It has been described that D2L receptor is mainly localized in the perinuclear region around the Golgi apparatus, whereas the short isoform is principally restricted to the plasma membrane and is therefore more accessible to DA agonists [31]. These observations suggest that DA effects on lactotropes, such as reduction of PRL secretion, inhibition of proliferation and induction of apoptosis, are mediated by the D2S receptor isoform.

Since p38 MAPK has been implicated in DA-induced apoptosis in neurons [19] and GH3 cells [20], we investigated involvement of this kinase in the apoptosis of lactotropes. DA- or CAB-induced apoptosis was reverted by a p38 MAPK inhibitor in anterior pituitary cells from primary cultures and in PR1-D2S cells, indicating that p38 MAPK is involved in the apoptosis of lactotropes induced by D2R activation. It has been reported that p38 and ERK are involved in DA-induced death in GH3 cells expressing D2Rs [20] and that ERK participates in the inhibition of cell proliferation in mice overexpressing D2S receptors [30]. Although p38 MAPK is involved in apoptosis of anterior pituitary cells, we cannot rule out the participation of other signaling pathways. We observed that DA phosphorylates p38 only in PR1-D2S cells. DA was shown to signal by different intracellular pathways in CHO cells transfected with D2L or D2S [33]. In addition, in GH4 pituitary cell line, D2S activation inhibits the phosphorylation of ERK induced by TRH whereas D2L activation failed to induce this action [34]. These data and our observations support the hypothesis that D2S and D2L receptor are functionally distinct in terms of coupling to MAPK pathways.

The first choice of treatment for prolactinomas are DA receptor agonists, being CAB the most effective one which inhibits PRL secretion and reduces tumor size [10], [35]. This drug suppresses PRL release in *in vitro* and *in vivo* rodent models [11], [12], and decreases cell viability in non-functioning human pituitary adenomas [36]. We previously reported that CAB induces apoptosis of lactotropes only when cells are cultured in the presence of E2 [2]. In this study, we show that administration of CAB to OVX rats increases apoptosis in the anterior pituitary gland only in E2-treated rats. These observations suggest that CAB's effect in reducing prolactinoma size involves induction of lactotrope apoptosis, and confirm that D2R activation induces apoptosis of anterior pituitary cells in an estrogen-dependent manner. We cannot discard that CAB induces apoptosis of non lactotrope cells. D2R is found in more than 75% of anterior pituitary cells, suggesting that non lactotrope cells express this receptor [37]. In fact, we reported that D2R activation by DA induces apoptosis of PRL- and non PRL-immunoreactive anterior pituitary cells [2].

E2 increases the expression of D2L mRNA in anterior pituitary cells [7]–[9]; however, we observed that D2S is the isoform involved in DA proapoptotic action. Therefore, our results suggest that E2 sensitizes anterior pituitary cells to DA by other mechanisms not involving D2R isoforms expression modulation. In fact, D2S expression in PR1 cells is not sufficient to induce apoptosis by DA, which requires E2-permissive action to produce this effect. This sensitization induced by E2 could be associated with changes in transduction pathways triggered by D2S activation. Whereas DA-induced apoptosis was prevented by a p38 MAPK inhibitor, D2S activation induced p38 phosphorylation, not only in the presence but also in the absence of E2. Considering that apoptosis induced by D2S receptor activation was only observed when cells were cultured in the presence of E2, the phosphorylation of p38 MAPK induced by DA seems to be a necessary but not a sufficient event to induce apoptosis of lactotropes. Our results show that E2 increases phosphorylation of p38 MAPK and suggest that this steroid enhances kinase activation induced by DA. p38 MAPK phosphorylation leads to activation of caspase 8 and 9, and proapoptotic proteins of the Bcl-2 family to trigger apoptosis via mitochondrial and extramitocondrial pathways [38]. We previously reported that E2 increases the ratio between proapoptotic and antiapoptotic proteins of the Bcl-2 family in the anterior pituitary [39]. In addition, since E2 increases p53 expression in this gland [40] and p53 was reported to be a target of p38 MAPK [41], the sensitization induced by E2 on anterior pituitary cells could involve an increment in p38 MAPK phosphorylation leading to p53 activation.

Prolactinomas are the most frequent pituitary tumors and medical therapy with D2R agonists is highly effective in most cases. However, some patients do not respond to pharmacological treatment [42]. D2R expression reduction and alterations in the intracellular pathways triggered by D2R activation have been associated with D2R agonist-resistance [10], [35]. In addition, differential expression of D2R isoforms may also be implicated in the resistance to these drugs. In fact, it has been reported that resistant prolactinomas have less expression of D2S mRNA than the responsive ones [14], [15]. It is also possible to speculate that low levels of estrogens in patients with hypogonadism induced by hyperprolactinemia may contribute to the development of pituitary adenomas and resistance to D2R agonist treatment. It would be interesting to study the relationship between circulating levels of estrogens in patients with prolactinomas and their response to DA agonist treatment.

Our study shows that the apoptosis of lactotropes induced by DA is mediated by D2S and is an estrogen-dependent process, involving p38 MAPK activation. These observations suggest that a decrease in D2S expression may participate in D2R agonist resistant prolactinomas.
